# Chronic Infection Drives Expression of the Inhibitory Receptor CD200R, and Its Ligand CD200, by Mouse and Human CD4 T Cells

**DOI:** 10.1371/journal.pone.0035466

**Published:** 2012-04-09

**Authors:** Stefano Caserta, Norman Nausch, Amy Sawtell, Rebecca Drummond, Tom Barr, Andrew S. MacDonald, Francisca Mutapi, Rose Zamoyska

**Affiliations:** Institute of Immunology and Infection Research, The University of Edinburgh, Edinburgh, United Kingdom; Federal University of São Paulo, Brazil

## Abstract

Certain parasites have evolved to evade the immune response and establish chronic infections that may persist for many years. T cell responses in these conditions become muted despite ongoing infection. Upregulation of surface receptors with inhibitory properties provides an immune cell-intrinsic mechanism that, under conditions of chronic infection, regulates immune responses and limits cellular activation and associated pathology. The negative regulator, CD200 receptor, and its ligand, CD200, have been shown to regulate macrophage activation and reduce pathology following infection. We show that CD4 T cells also increase expression of inhibitory CD200 receptors (CD200R) in response to chronic infection. CD200R was upregulated on murine effector T cells in response to infection with bacterial, *Salmonella enterica,* or helminth, *Schistosoma mansoni,* pathogens that respectively drive predominant Th1- or Th2-responses. *In vitro* chronic and prolonged stimuli were required for the sustained upregulation of CD200R, and its expression coincided with loss of multifunctional potential in T effector cells during infection. Importantly, we show an association between IL-4 production and CD200R expression on T effector cells from humans infected with *Schistosoma haematobium* that correlated effectively with egg burden and, thus infection intensity. Our results indicate a role of CD200R:CD200 in T cell responses to helminths which has diagnostic and prognostic relevance as a marker of infection for chronic schistosomiasis in mouse and man.

## Introduction

Among parasitic diseases, schistosomiasis is a major cause of morbidity in the developing world with high prevalence and serious disease-associated disability and socio-economic impact [1], [2]. In endemic areas the burden of helminth infection is frequently mixed [3], with a small proportion of individuals suffering much higher infection loads than others. Children generally lack adaptive immunity to schistosomes and are more likely to accumulate substantial worm burden resulting in diminished physical fitness and impaired development [4]. How effective anti-helminth immunity is developed and maintained is still unknown and early prognostic tools to distinguish individuals more likely to progress into severe disease are needed.

Recently, it has become clear that helminths modulate the balance between effector and immunoregulatory mechanisms during infection [5]. Furthermore re-exposure to helminth-derived antigens (Ag) induces T cell anergy [6], potentially undermining pathogen elimination, yet it remains undetermined whether anergy is a risk factor for disease severity in schistosomiasis. Indeed, immune responses must be initiated and terminated appropriately to maintain peripheral tolerance and immune homeostasis. In this context, T cell suppression/anergy might be beneficial to limit immune-mediated damage and fibrosis in self-tissues in severe schistosomiasis.

Potentially self-damaging peripheral T cell responses are regulated by cell extrinsic mechanisms, such as the induction of T regulatory cells and intrinsically via the upregulation of surface inhibitory receptors. The latter include the CD28-superfamily members CTLA-4, ICOS and PD-1 [7], which are expressed on activated T cells during immune responses. Interaction of these receptors with their ligands: CD80/CD86, B7H2 and PD-L1/PD-L2, regulates T cell activity [8], [9], [10], [11]. Another inhibitory receptor:ligand pair, CD200 receptor (CD200R):CD200 (OX-2), has so far been studied mainly in myeloid cells [12]. CD200R:CD200 interaction delivers inhibitory signals, downregulating the activity of CD200R bearing cells [13]. CD200R expression has been reported primarily in myeloid lineage subsets such as: macrophages, dendritic cells (DC), mast cells, neutrophils and basophils [14], [15], while CD200 is more widely expressed on T and B cells, DC, endothelium and neurons [16], [17]. CD200 deficient (*CD200^–/–^*) mice display increased susceptibility to T cell-mediated autoimmune diseases [18] and have greater sensitivity to influenza infection, largely due to a failure in regulation of airway macrophages [19]. Enhanced pathological T cell responses were also reported in influenza infected *CD200^–/–^* mice [20]. Thus, CD200R:CD200 interactions are important in immune regulation and pathology. However, to date any suggestion that CD200R:CD200 influences T cell responses has largely come from tumor studies [21], [22] and it remains largely unknown how CD200R and CD200 are regulated in T cells during infection.

Expression of CD200R [14], [15] and CD200 [17], [18] on subpopulations of human and mouse peripheral T cells was reported previously and CD200 expression increased in activated T cells *in vitro* [19], [23]. Similarly, high expression of CD200R was detected in more differentiated, central and effector memory T cells [14] and was particularly apparent in polarized Th2 cells [15]. Thus, we sought to investigate the upregulation of CD200 and, particularly, CD200R on T cells during persistent Ag exposure. We show that chronicity of Ag stimulation influenced both CD200 and CD200R expression by mouse T cells under either Th1 or Th2 culture conditions. Furthermore, during sustained Ag-exposure *in vivo*, CD200R expression increased in Th2 (*Schistosoma mansoni*) and Th1 (*Salmonella enterica*) infection models. Finally, we found significant correlation between infection intensity and CD200R expression by Th2 prone cells from *Schistosoma haematobium* infected humans. Our results show that chronic Ag exposure results in CD200/CD200R upregulation on T cells irrespective of the cytokine milieu. Importantly, our data indicate that CD200R expression by CD4 T cells is a useful indicator of infection intensity and T cell function in areas of endemic schistosomiasis. Thus analysis of CD200R expression on T cells may facilitate early diagnosis, the evaluation of disease progression and the impact of pharmacological interventions on these neglected diseases.

## Results

### Sustained TCR-signals Drive Co-expression of CD200 and CD200R in T Cells

**Figure 1:**
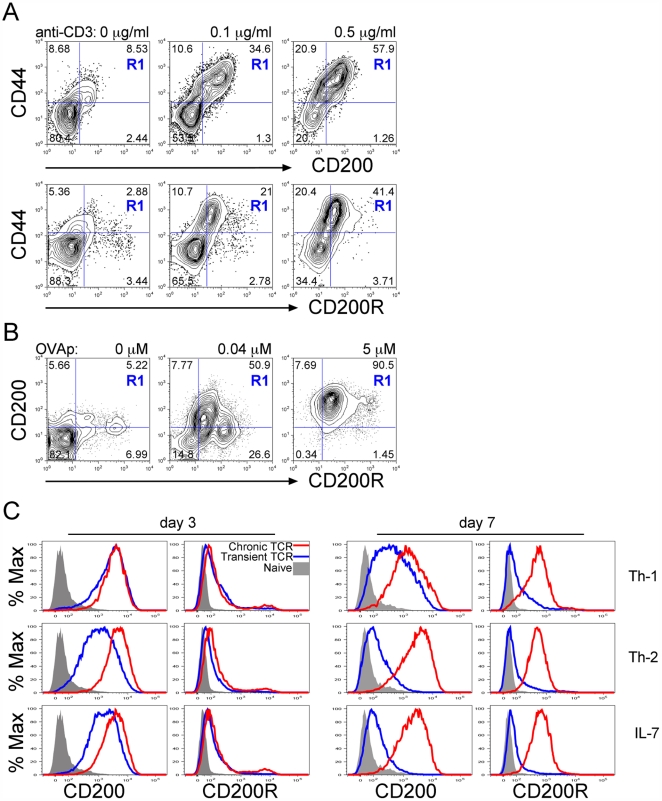
T cells co-express CD200 and CD200R following TCR activation. A. Naïve C57BL/6 peripheral LN cells were cultured with a titration of anti-CD3 + anti-CD28 (2 µg/ml) for 3d. Contour plots show upregulation of CD200 (upper row) and CD200R (lower row) together with CD44 (R1) in gated CD4 cells. B. DO11.10 LN and spleen cells were cultured with a titration of OVA peptide (ISQAVHAAHAEINEAGR) for 3d. CD200R:CD200 co-expression (R1) is shown in gated CD4 cells. C. Naïve LN cells were stimulated with anti-CD3 (1 µg/ml) + anti-CD28 (2 µg/ml) either transiently on Ab-coated wells for the first 2d then removed to fresh media (blue lines) or chronically with Ab-coated aAPC present throughout the culture (red lines) in Th1 (top row), Th2 (middle row) or non-polarising (IL-7, bottom row) conditions. Histograms show expression of CD200 and CD200R in gated CD4 cells at d3 (left) and d7 (right) compared to naïve levels (grey filled histograms). Data are representative of 6 independent experiments and statistical validation is shown in Figure S1A and S3C.

Activated T cells upregulated CD200 *in vitro* [23] and memory T cells expressed more CD200R *ex vivo* than their naive counterparts [14], yet it was unclear whether T cells co-expressed both CD200 and CD200R, and whether upregulation of these molecules was influenced by the strength of TCR stimulation. We characterised the expression of CD200 and CD200R on T cells following TCR activation *in vitro*. Lymph node (LN) cells from naïve mice were stimulated with a titration of anti-CD3 mAb together with anti-CD28 mAb for 3 days. CD4 T cells upregulated CD44, a stable marker of activation, together with CD200 and CD200R in a dose dependent manner (Figure 1A, R1; Figure S1A). Similarly DO11.10 TCR transgenic cells upregulated and co-expressed CD200 and CD200R in response to increasing doses of antigenic peptide (Figure 1B, R1). Neither CD200 nor CD200R were upregulated on T cells cultured in IL-7 (Figure S1A), which promotes naïve cell survival without activation [24].

We asked whether expression of CD200R and CD200 was affected by either the duration of TCR stimulation or additional cytokine signals provided in Th1, Th2 and non-polarising (IL-7) conditions. Naïve CD4 T cells were transiently stimulated (48h) with anti-CD3 and anti-CD28 mAb-coated wells before removal to fresh medium for the remainder of the culture. Alternatively, sustained, chronic TCR stimulation was provided by continuous culture with artificial antigen presenting cells (aAPC) coated with anti-CD3 and anti-CD28 mAb [25]. At d3 both of these stimuli elicited comparable activation of the T cells as judged by CD25 and CD44 upregulation (Figure S1B). Under Th1 polarising conditions, activated CD44^hi^ CD25+ T cells were shown to express CD200 and this expression was maintained until d7 in both transiently and chronically stimulated cells. Transient stimulation in Th2 or neutral conditions provoked less upregulation of CD200 at d3, and by d7 levels had dropped close to background. Sustained stimulation produced comparable and durable expression of CD200 regardless of polarising environment.

In contrast, modest upregulation of CD200R was observed by d3 in any culture conditions (Figure 1C). By d7 more extensive upregulation of CD200R occurred, but only after chronic stimulation (Figure 1C and S2A). In all cases T cells up-regulated both CD200 and CD200R compared to naïve CD4 T cells (Figure 1C) and the two molecules were co-expressed by the majority of the cells upon chronic stimulation (Figure S2A). In addition both molecules were co-expressed on activated CD44^+^CD25^+^ T cells (Figure S2B). Thus the key determining factor influencing CD200R expression was the chronicity of the TCR stimulation, whereas CD200 expression was influenced both by sustained stimulation and the polarising environment.

### Multifunctional Potential is Decreased in CD200R Expressing CD4 T Cells

CD200R, but not CD200, delivers inhibitory signals at least in myeloid lineage cells, therefore we investigated CD200R expression during T cell differentiation. Activated T cells initially secrete multiple cytokines, for example, IL-2, TNFα and IFNγ [26]. However, upon chronic Ag exposure, which induces terminal differentiation of CD4 T cells into short-lived Th1 [26], [27] or Th2 [28] effector cells, the multifunctional potential of T cells becomes exhausted together with their proliferative potential.

**Figure 2:**
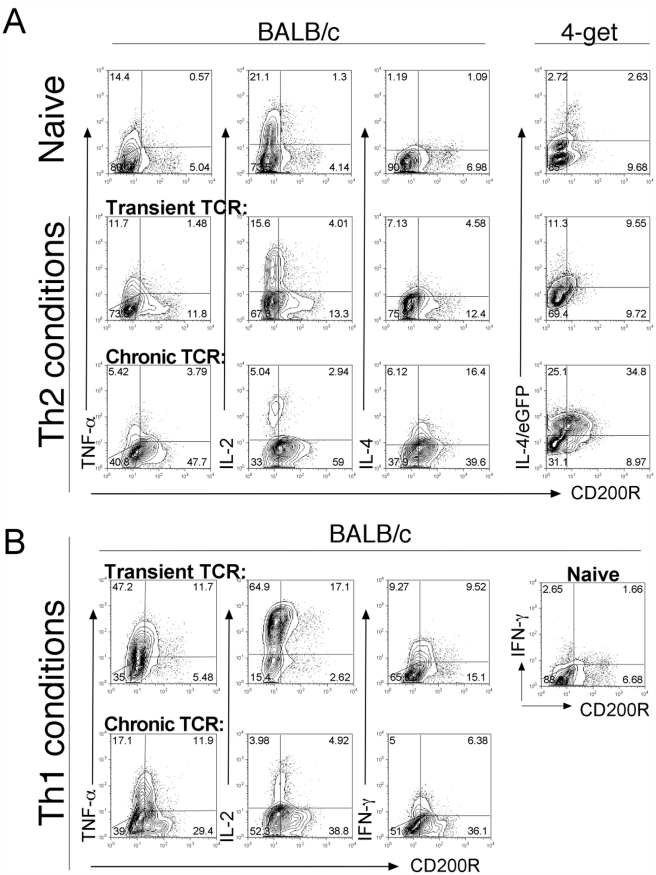
Progressive acquisition of CD200R correlates with effector cytokine secretion. Peripheral LN cells from BALB/c mice and 4-get mice were analysed ex vivo (Naive) or after culture for 7d in transient TCR or chronic TCR stimulation conditions, as indicated, using polarising culture conditions to promote Th2 (A) or Th1 (B) cytokine differentiation. Intracellular cytokine staining with CD200R expression is shown for TNFα, IL-2, IL-4 and IFNγ. Intracellular staining with anti-GFP was used to enhance eGFP signal in 4-get cells (control stainings for anti-GFP are shown in Figure S5B & C). Contour plots show percentage of cytokine^+^ gated CD4 cells; gates were based on unstimulated controls (Figure S3A and S3B). Data are representative of 5 independent experiments. Pairwise comparison of multifunctional cytokine loss from replicate cultures is shown in Figure S3D-F.

CD4 T cells expressed low levels of CD200R during the first three days of stimulation and mainly produced IL-2 and TNFα irrespectively of the polarising conditions in the culture (data not shown). By d7 transiently stimulated Th2 cells were still largely CD200R negative and continued to produce multifunctional cytokines, TNFα and IL-2, similar to naïve T cells. In contrast, chronically stimulated cells upregulated CD200R and lost the ability to secrete TNFα and IL-2 (Figure 2A-B and Figure S3E-F). Concomitant with the loss of multifunctional cytokine production, in Th2 culture conditions, a substantial fraction of the chronically stimulated cells produced IL-4, or IL-4 mRNA as measured by eGFP expression in 4-get T cells (Figure 2A) [29]. Similarly under Th1 polarised culture conditions (Figure 2B), the potential to secrete TNFα and IL-2 was also decreased following chronic TCR stimulation, which favoured the upregulation of CD200R expression (Figure 2B and Figure S3C-D). These data support the hypothesis that *in vitro* CD200R expression is acquired by cells that have decreased multifunctional cytokine potential.

### Chronic Schistosomiasis Leads to CD200 and CD200R Co-expression in T Cells

Sustained TCR stimulation induced CD200R:CD200 co-expression in T cells *in vitro* (Figure 1) but it was unknown whether this might similarly occur *in vivo*. We infected mice with *Schistosoma mansoni* cercariae [30] which develop into adult worms and by 6–8 weeks post-infection continuously produce eggs which provide persistent systemic Schistosome Egg Ags and drive a chronic Th2 response [5]. At week 8 post-infection, we analysed CD200 and CD200R expression in T cells from the mesenteric LN (MesLN) together with CD44 to distinguish Ag-primed (CD44^hi^) from naïve (CD44^lo^) cells [31].

**Figure 3:**
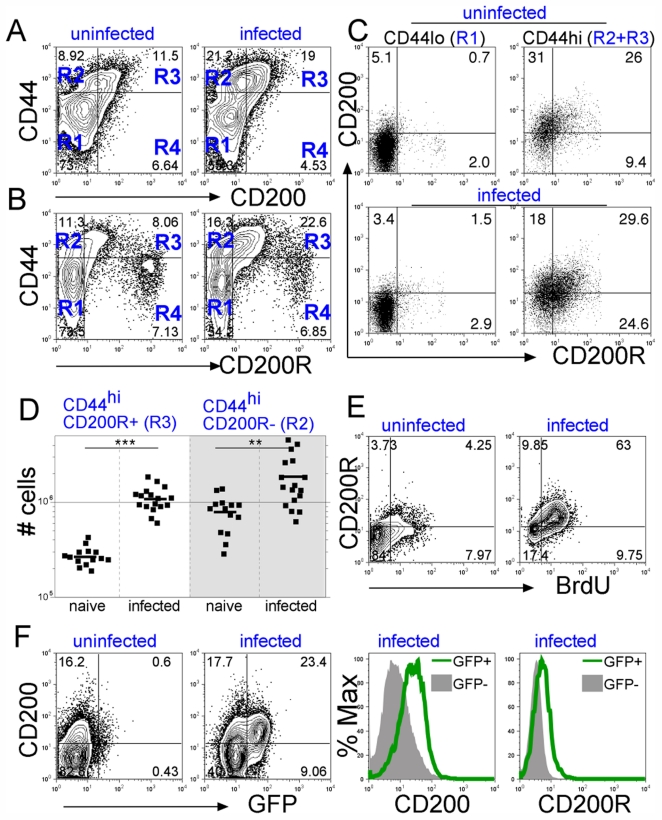
Chronic infection induces CD200 and CD200R expression in CD4 T cells. C57BL/6 (A–E) and 4-get (F) mice were infected with *S. mansoni*; MesLN (A–D, F) and spleen (E) were analyzed. A. Representative contour plot of CD44 with CD200, and B. CD44 with CD200R is shown for gated CD4 T cells. Upon infection (8 wk), CD44^hi^CD200^+^CD4 cells (panel A, R3) increased from 7.71±2.66% (n = 14) to 15.2±3.72% (n = 17, p<0.0001); CD44^hi^CD200R^+^CD4 cells (panel B, R3) increased from 4.36±1.69% (n = 14) to 15.3±4.67% (n = 17, p<0.0001). Data were pooled from 4 independent experiments. C. Dot plots show CD200 and CD200R co-expression in CD44^lo^ and CD44^hi^ CD4 cells from uninfected and infected mice. D. Graph shows absolute numbers of CD200R^+^ (panel B, R3) and CD200R^–^ (panel B, R2) CD44^hi^CD4 cells from 4 independent experiments. Upon infection CD44hiCD200R+ cells increased from (2.7±0.6)× 10^5^ (n = 14) to (10.8±3.2)×10^5^ (n = 17, ***p = 3.03×10^–10^), while CD44^hi^ CD200R^–^ cells increased from (7.9±3.3)×10^5^ to (18.6±11.9)×10^5^ (**p = 0.003). E. Contour plots show CD200R expression in proliferating (BrdU^+^) cells, in gated CD4 cells for spleen of uninfected and infected mice. Upon infection BrdU^+^CD200R^+^ CD4 T cells increased from 4.5±0.69 (n = 4) to 46.6±17 (n = 10) p<0.0005. Specificity controls for BrdU staining are shown in Figure S7A & B. F. Contour plots (left) show CD200 and eGFP expression; CD200^+^GFP^+^CD4 cells increased from 0.64±0.12% to 13.7±7.24% after infection (p = 0.01, n = 4). Histogram overlays (right) show CD200 and CD200R levels in gated GFP^+^ (green line) and GFP^–^ (filled histograms) CD4 cells from infected mice. Control stainings for intracellular GFP staining are shown in Fig S5B & C. ** = p<0.0001, * = p<0.001. Results are representative of at least 4 (A–D) and 3 (E-F) independent experiments (n = 3 mice/group).

In uninfected control mice the majority of CD4 T cells were of naive CD44^lo^ phenotype (R1, Figure 3A and 3B), with the remainder showing a memory/effector (CD44^hi^) T cell phenotype, consistent with exposure to environmental Ags (Figure 3A, R2+R3 and Figure S4A). CD200 was essentially absent from the CD44^lo^ population (Figure 3C, left panels, R1) but was expressed on approximately half of the CD44^hi^ T cells (Figure 3C, right panels, R2+R3). Infected mice doubled their percentage of CD44^hi^ T cells (Figure S4A) and the CD44^hi^ T cells from infected mice had a similar proportion of CD200^+^ cells (Figure 3C). This indicates that CD200R:CD200 expression is only acquired by antigen experienced T cells, whether these are from naïve mice exposed to environmental antigens or from infected mice. Staining for CD200R and CD44 detected three distinct cell populations in MesLN that varied in their intensity of staining for CD200R (Figure 3B). The first were naive CD44^lo^ cells, which were CD200R^–^ and mostly CD200^–^ (Figure 3C, left panels, R1). The second were CD44^hi^ cells which expressed intermediate CD200R levels and were also largely CD200^+^ (Figure 3C, right panels, R2+R3). These cells nearly trebled in frequency among CD44^hi^ CD4 T cells following infection (Figure 3B, R3). The third, which were in the CD44^lo^ quadrant, expressed very high CD200R levels (Figure 3B, R4) and were identified as early apoptotic/necrotic cells (Figure S5A). The proportion of these CD44^lo^ CD200R^+^ cells did not change with infection (Figure 3B, R4) and were excluded from subsequent analysis. Thus, with infection an increased fraction of the total activated T cells (CD44^hi^) upregulated and co-expressed CD200R and CD200 (Figure 3C and Figure S4B).

In absolute numbers, CD44^hi^CD200R^+^ and CD44^hi^CD200R^–^ cells expanded 4- and 2.4-fold respectively during infection (Figure 3D), suggesting that CD200R^+^ cells significantly outgrow other activated cells in chronic schistosomiasis. BrdU administration *in vivo* showed that CD200R^+^ CD4 T cells in spleen and lymph node had proliferated upon infection (Figure 3E and S6). Egg-derived Ag drives a strong Th2 response [6] and Th2-skewed eGFP^+^ CD4 T cells increased in infected 4-get mice compared to controls. Importantly, these eGFP^+^ effectors expressed higher levels of CD200 and CD200R than their eGFP^–^ counterparts (Figure 3F). Together these results indicate that, during chronic helminth infection, CD200R and its ligand CD200 are upregulated and co-expressed in chronically activated CD4 T cells.

### CD200R Expressing T Cells Lose Multifunctional Potential during Chronic Infection

**Figure 4:**
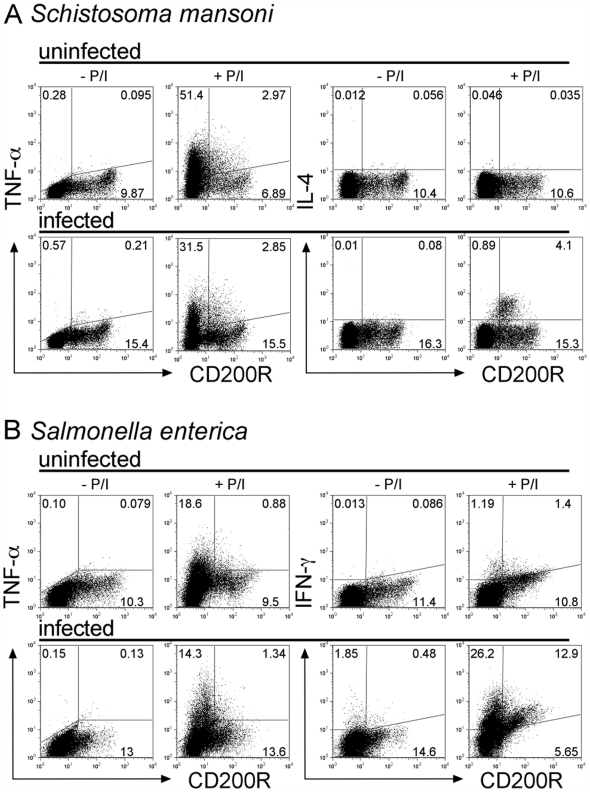
Effector cytokine potential is acquired alongside CD200R expression in vivo. Dot plots show cytokine^+^ gated CD4 cells *ex vivo* (-P/I) and after recall with pdbU + Ionomycin (+P/I, 5h); gates are based on unstimulated controls (-P/I). A. Infection with *S. mansoni* (8wk) increased CD200R^+^ IL-4^+^ CD4 cells in MesLN from 0.017±0.019% to 3.39±1.36% and 83±4.14% of IL-4^+^ cells were CD200R^+^, while CD200R^–^ TNFα^+^ CD4 cells decreased from 47.5±4.66% to 30.0±0.99%. (n = 3, p = 0.01). B. Infection with *S. enterica* increased IFNγ^+^ CD4 cells in spleen from 1.9±0.65% to 31.1±4.4% and 49.5±9.86% of IFNγ^+^ cells were CD200R^+^, while CD200R^–^ TNFα^+^ CD4 cells decreased from 19.6±2.31% to 15.1±1.56%. (n = 5, p = 0.01). Results are representative of at least 3 (A, n = 3 mice/group) and 2 (B, n = 5 mice/group) independent experiments.

We sought to investigate whether multifunctional cytokine secretion had been lost in CD200R^+^ effectors generated during infection, by concomitant analysis of CD200R and cytokine expression. In both uninfected and *S. mansoni* infected mice a proportion of CD4 T cells produced TNFα but these cells were primarily CD200R^–^ and decreased upon infection (Figure 4A and S8A). In contrast, IL-4^+^ cells from infected mice were almost exclusively CD200R^+^ (Figure 4A). Thus chronic infection with *S. mansoni* led to differentiation of Th2 effectors with decreased multifunctional potential, which were IL-4^+^, CD200R^+^.

CD200R expression was previously reported in Th2, but not Th1 subsets [15], and yet we found that Th1 cells also acquired CD200R expression *in vitro* (Figures 1 and 2). Infection with *Salmonella enterica* is controlled by CD4 T cells making a predominantly Th1 response which persists for several weeks [32]. We investigated whether this chronic Th1 stimulation also led to upregulation of CD200R on IFNγ^+^ Th1 effectors *in vivo*, and found this was the case (Figure 4B). As before (Figure 2 and 4A), CD4 TNFα producing cells from uninfected or *S. enterica* infected mice were mainly CD200R^–^ and decreased during infection (Figure S8B). On the other hand, infected animals had IFNγ^+^ CD4 cells, some of which upregulated CD200R. Interestingly, and unlike IL-4^+^ effector cells, ∼50% IFNγ^+^ cells remained CD200R^–^. Together these data suggest that the expression of CD200R on CD4 T cells relates to persistent Ag exposure rather than reflecting a particular helper subset.

### The Intensity of Chronic Human Schistosomiasis Correlates with CD200R Expression in Effector T Cells

**Figure 5:**
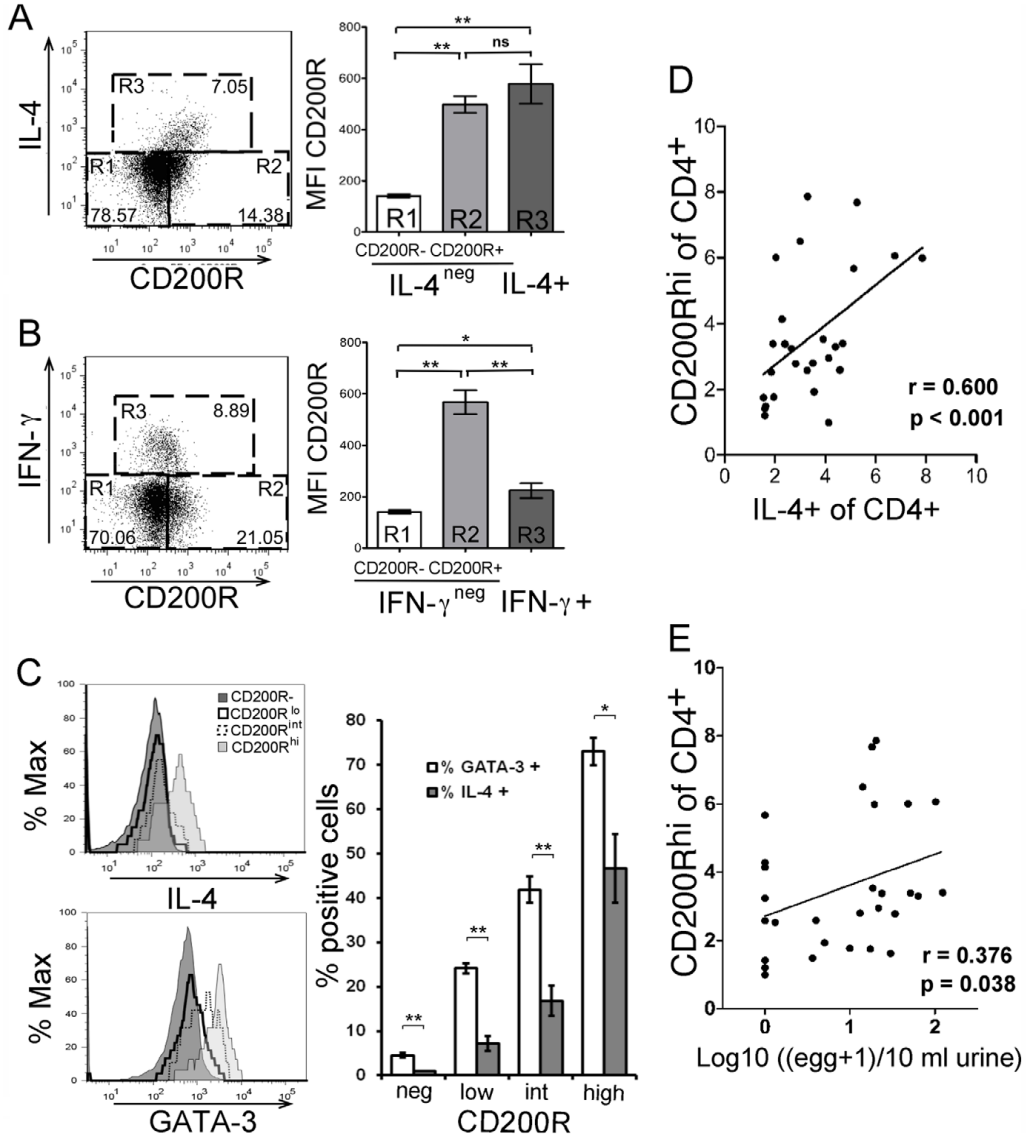
CD200R correlates with infection intensity in human schistosomiasis. Expression of CD200R and IL-4 (A) or IFNγ (B) in gated CD4 T cells from blood after recall with PMA + Ionomycin. Isotype control stainings are shown in Figure S9. Charts show mean (±s.e.) of CD200R MFI in R1-3. C. CD200R-, CD200R^lo^ and CD200R^hi^ αβTCR^+^CD4 cells were analysed for IL-4 and GATA-3 expression. One representative sample is shown in left histograms. Mean percentage (±s.e.) of cells in each population are shown in the bar graph. * = p<0.05, ** = p<0.01 (n = 8, ANOVA A–C). D. Correlation between the percentages of IL-4^+^ and CD200R^+^ CD4 T cells, respectively determined by intracellular and surface stain. E. Correlation between the proportions of CD200R^hi^CD4 T cells and infection intensity determined as urine egg counts (n = 29, D-E). Percentages of CD200R^hi^CD4 T cells and IL-4^+^CD4 T cells were arcsine square root transformed to allow the use of parametric tests. Infection intensity was Log10(x+1) transformed. To allow for potential confounding effects of sex (categorical variable) and age (continuous variable) transformed data were assessed by ANOVA. Resulting residuals were used to analyse the correlation between both CD200R^hi^CD4 T cells and IL-4+CD4 T cells or between CD200R^hi^CD4 T cells and infection intensity of which the r-correlation coefficients and p-values are indicated.

Distinct CD200R isoforms with poorly defined roles have been identified in humans and mice, introducing the possibility that differences in the CD200R:CD200 axis might exist between species. CD200R mRNA expression was reported in human Th2 clones *in vitro* [15] and human memory T cell subsets differentially expressed CD200R, although the reasons for such differences remain unknown [14]. We investigated CD200R expression in PBMC from individuals endemically exposed to *Schistosoma haematobium,* the cause of urinary schistosomiasis. In agreement with a previous study using healthy subjects [14], CD200R expression was limited to memory/activated CD45RO^+^ or CD45RA^–^ CD4 T cells (Figure S9A). We analyzed cytokine expression alongside CD200R expression in activated CD4 cells from infected individuals. IL-4^+^ CD4 T cells were almost exclusively CD200R^+^ (Figure 5A). Comparison of the MFIs showed that IL-4^+^ cells had the highest expression of CD200R (Figure 5A, bar graph). Interestingly, IFNγ^+^ T cells from the same individuals expressed significantly lower levels of CD200R (Figure 5B, R3) with an MFI close to that of the CD200R^–^ population (Figure 5B, R1). Some cells were CD200R^+^ (Figure 5A and B, R2) but produced neither IL-4 nor IFNγ suggesting they might be Th2-lineage cells refractory to restimulation *in vitro*. We looked for expression of the signature Th2 transcription factor, GATA-3, and found that the proportions of GATA-3^+^ cells within the CD200R^+^ cells were higher than proportions of IL-4^+^ cells, confirming that there are more Th2 lineage cells within CD200R^+^ CD4 T cells than indicated by IL-4^+^ cells (Figure 5C). Examination of multiple individuals (n = 29) showed significant linear correlation between IL-4^+^ CD4 effectors and CD200R expression by CD4 cells (Figure 5D).

Finally, since CD200R expression in mouse depended on chronic Ag exposure, we investigated the association between CD200R^hi^ CD4 T cells and infection intensity, and thus Ag load, in a cohort (n = 29, 6–16 years) of young individuals which included a number of healthy controls (n = 8). At the time of blood sampling, individual infection burden was measured as urinary schistosomal egg counts (average 21.5eggs/10ml). Potentially confounding effects of sex and age on the analysis notwithstanding, there was a significant correlation between infection intensity and the proportions of CD200R^hi^ CD4 T cells in blood (Figure 5E). These results indicate that *S. haematobium* infection intensity influences CD200R expression in human CD4 T cells and suggests that CD200R expression has potential as a biomarker for parasite load.

## Discussion

Expression of CD200R and CD200 by T cells has been reported previously [14], [15] but has not been correlated with antigen exposure *in vivo*. We showed that the inhibitory receptor:ligand pair of molecules, CD200R:CD200, was upregulated on CD4 T cells during persistent infection. Receptor and ligand were co-expressed on individual cells and prolonged expression of CD200R, in particular, required sustained Ag stimulation. A fraction of the activated, CD44^hi^ T cells generated in chronic helminth infection upregulated CD200R and these cells were specifically increased in expression of IL-4/eGFP. Indeed, acquisition of CD200R paralleled loss of multifunctional potential together with differentiation to effector cytokine secretion. Importantly, infection intensity positively correlated with CD200R expression by CD4 T cells in individuals endemically exposed to schistosomal Ag. These data indicate that CD200R is a novel marker that may be used to monitor immune responses to helminths in both mouse and man, with implications for evaluating immunopathology and for assessing the success of immunotherapies for these debilitating diseases.

CD200 has a short intra-cytoplasmic tail devoid of known signalling motifs hence it is unlikely to signal [12], mainly acting as ligand for CD200R. However, co-expression of both receptor and ligand on T cells introduces the possibility that these cells might negatively regulate each other, since CD200 on T cells could bind CD200R either in cis or trans, potentially inducing an immunoregulatory signal in CD200R^+^ T cells and/or myeloid-origin targets. Further studies are required to validate these hyphotheses. Interestingly CD200^–/–^ mice suffer severe T cell mediated immune pathology upon viral infection [19], implying that physiological expression of CD200 exerts immunoregulatory control over peripheral immune responses. Treatment with CD200 agonistic fusion proteins ameliorated lethal immune reactions during influenza infection, suggesting that the immunoregulatory balance of CD200 is relevant in pathological conditions [19]. CD200 expression is relatively diffuse in epithelial and smooth muscle cells and it is pertinent that CD200 expression is limited to certain cell types in lymphoid tissues. We show that expression of CD200 in activated T cells in lymphoid tissues changes substantially with chronic infection, thus increasing the potential immunosuppressive milieu via CD200R:CD200 interactions.

What is the biological relevance of CD200R:CD200 expression in effector/memory T cells generated in chronic infection? The immunoregulatory signals downstream of CD200R engagement have been characterized in myeloid cells [13], [33] but whether the same signaling cascades are activated in T cells and potentially integrate with TCR signals to shape lymphocyte fate remain unknown. At the time of clonal expansion, CD200R might influence T cell differentiation and, thereafter, exhaustion. In support of this idea we found that multifunctional T cells which secrete non-polarizing cytokines (i.e. TNFα and IL-2) expressed lower amounts of CD200R while fully polarised effector T cells acquired CD200R. Following helminth infection activated CD200R^+^ CD4 T cells accumulated substantially and contained IL-4 secreting effector cells. In human samples, higher CD200R expression correlated positively with IL-4 secretion and GATA-3 expression. Interestingly, whereas all eGFP^+^ CD4 T cells during schistosomiasis preserved CD200R, only a proportion secreted IL-4 indicating that not all Th2-primed cells acquired/retained effector function. A similar situation was observed in humans, where CD200R^+^GATA-3^+^ cells outnumbered CD200R^+^IL-4^+^ cells (Figure 5C). Thus, we suggest that a substantial fraction of CD200R^+^ CD4 T cells might become hyporesponsive as a consequence of chronic Ag exposure in mice and humans. Indeed, we found that CD200R warrants further investigation as a biomarker for *S. haematobium* infection intensity as it was considerably better than that seen with other biomarkers, such as parasite-specific IL-4 secretion [34] or systemic levels of IL-4 [35], which are more dependent on the functional activation of effectors, and more likely to be impaired in chronic infections.

CD200R expression was previously correlated to Th2-subset differentiation [15]. However, our data suggested that CD200R:CD200 expression was not limited to Th2 conditions, but rather may be more generally involved in chronic Ag exposure. CD200R was upregulated in both Th1 and Th2 cells *in vitro* and during *S. mansoni* and *S. enterica* infections. Similarly in *S. haematobium* infected humans, we found that CD200R expression was significantly increased in Th2-prone cells. However IFNγ^+^ cells from the same individuals also showed low but significant expression of CD200R. Although we show that CD200R is not limited to Th2 T cells, the regulation of CD200 and CD200R between Th1 and Th2 cells may differ depending on the TCR signal strength necessary for their differentiation. CD200∶CD200R expression has been linked previously to the control of autoimmunity [18] and to immune evasion by tumour cells [21]. We show here that they are also relevant in CD4 T cell response to persistent infections. These data raise the possibility that these molecules will be valuable markers of infection intensity in chronic helminth infections and they may have potential as therapeutic targets.

## Materials and Methods

### Mice and Infections

C57BL/6, BALB/c, DO11.10 and 4-get (IL-4-eGFP) mice were bred at the University of Edinburgh (UK). DO11.10 mice are transgenic for the OVA_323–339_ peptide-specific TCR restricted by I-A^d^ on a BALB/c genetic background [36], while 4-get mice have an IRES-eGFP reporter knocked into the IL-4 locus and report the presence of IL-4 mRNA and protein [29]. Maintenance and experimentation of animals were carried out under guidelines overseen by the University of Edinburgh Biological Services Ethical Review Panel Application PL26-07 and the UK Home Office.

*Biomphalaria glabrata* snails infected with *S. mansoni* were obtained from Dr. Fred Lewis (NIAID Schistosomiasis Resource Center at Biomedical Research Institute, Rockville, MD). Mice were infected percutaneously (80-180 cercariae) as described [30]. Mice received BrdU (i.p. and orally) 4d before analysis and incorporation was determined as described [37], [38].

The *aro*A attenuated strain of *S. enterica* serovar Typhimurium, SL3261, was used in infections [39], as previously described (10^6^ CFU/mouse, i.v.) [40]. In spleen, bacterial loads were typically 2–5×10^5^ CFU/g.

### Study Population and Human Patients

Blood samples were obtained from residents in Mashonaland East Province of Zimbabwe (31o91'E; 17o63'S), a Schistosoma haematobium endemic region. Permission for study execution in the area was obtained from the Provincial Medical Director while institutional approval was obtained from the University of Zimbabwe. Ethical approval for the study was obtained from the Medical Research Council of Zimbabwe. Participants are enrolled in an ongoing immuno-epidemiology study of human schistosomiasis, never underwent anti-helminthic treatment prior to this study and received anti-helminth drug, praziquantel (40 mg/kg) at the end of the study. Study aims and procedures were explained to participants and their parents/guardians in the local language prior to enrollment. Written consent was obtained from all participants or their parents/guardians in case of children. Participants were free to leave the study at any time during the study.

### Mouse T Cell Primary Cultures

Peripheral lymph node cells were pooled from axillary, inguinal, brachial, and cervical lymph nodes (excluding mesenteric (mes)LN). Where explicitly stated in the figure legends mesLN were collected. After lymphoid organ disaggregation, cells were washed and resuspended in complete media (RPMI-1640, 5%FCS, 2 mM L-gln, 100 U/ml penicillin/streptomycin, and 2.5×10^–5^ M 2β-ME (Invitrogen)). Anti-CD3 (Clone 145-2C11, BD) at 0-1 µg/ml and anti-CD28 (Clone 37.51, BD) at 2 µg/ml mAb were coated on plates (1h, 37°C, in PBS) and washed twice. In transient TCR stimulations, cells were moved from Ab-coated to fresh wells on d2. aAPC were described previously [25]. aAPC:CD4 cell ratios in culture were 2.5∶1. Polarising culture conditions were: Th1: IL-12 (Peprotech, 5 ng/ml) and anti–IL-4 mAb (11B11, BD); Th2: IL-4 (Peprotech, 5 ng/ml) and anti-IL-12 mAb (C17.8, BD) and non-polarising cultures: IL-7 (Peprotech, 50 ng/ml). Blocking mAb (2.5 g/ml) were added on d0 and d3.

### Flow Cytometry

Mouse cells were incubated with anti-FcR blocking mAb (Biolegend) for 10 min, at RT and stained (15 min, 4°C) with PE-TexasRed- or PerCP-labeled anti-CD4 (BD) and PE-labeled anti-CD44 (IM7, eBioscience), APC-labeled anti-CD200 (eBioscience), FITC-labeled anti-CD200R (Serotec) mAb. Human PBMC were isolated (Lymphoprep™) and cryo-preserved. Following thawing, cells were resuspended in complete media, washed twice, counted (Trypan Blue) and stained with Alexa-488-labeled anti-CD4 (OKT-4), PE-labeled anti-CD200R (Ox108), v500-labeled anti-CD8 (from eBioscience) with Pacific blue-labeled anti-αβTCR (IP26, Biolegend). Isotype control staining for CD200 and CD200R Abs are shown in Figure S7 (mouse) and Figure S9 (human).

Intracellular cytokine staining: 5×10^5^ T cells were incubated with PerCP-labeled anti-CD4 and FITC-labeled anti-CD200R mAb and stimulated with PdbU (70 ng/ml), Ionomycin (1 µg/ml) and BrefeldinA (5 µg/ml) for 5 h at 37°C. After fixation in 2% isotonic formaldehyde and permeabilization in buffer containing PBS 2%FCS, 0.2%NaN_3_, 0.5% saponin, 2% rat serum, cells were stained with anti–IL-2, anti–IL-4 (both BD), anti–IFNγ, anti–TNFα (both eBioscience) and anti-GFP mAb (Invitrogen) for 30min at RT. Cells were washed twice in permeabilization buffer. Human PBMC were incubated for 4 h at 37°C with PMA (10 ng/ml), Ionomycin (1 µM) and GolgiStop^TM^, washed, and stained for surface markers as described above, then permeabilized and stained with PE-Cy7-labeled anti-IL-4 and anti-IFNγ mAb (eBioscience). Cells were washed in staining buffer before acquisition on FACSCalibur™ or LSR II cytometers (BD). Data were analyzed in FlowJo software (TreeStar, Ashland, USA).

### Statistical Analyses

Unpaired two-tailed t-test was used in mouse studies, unless indicated in the Figure legend that paired t-test was used. SPSSv14 software was used for analysis of human data [41]. In the human studies, percentages of CD200R^hi^CD4 T cells and IL-4^+^CD4 T cells were arcsine square root transformed to allow the use of parametric tests. Infection intensity was Log10(x+1) transformed. To allow for potential confounding effects of sex (categorical variable) and age (continuous variable) transformed data were assessed by ANOVA. Resulting residuals were used to analyse the correlation between both CD200R^hi^CD4 T cells and IL-4+CD4 T cells or between CD200R^hi^CD4 T cells and infection intensity of which the r-correlation coefficients and p-values are indicated.

## Supporting Information

Figure S1 **T cells co-express CD200 and CD200R following TCR stimulation.** A. Naïve C57BL/6 peripheral LN cells were cultured with a titration of anti-CD3 + anti-CD28 (2 µg/ml) (white bars) or only with recombinant mouse IL-7 (10 ng/ml, black bar) for 3d. Bar graphs show average±SEM Mean Fluorescence Intensity (MFI) expression of CD200 (left) and CD200R (middle) in CD4 T cells and percentage of CD200^+^ CD200R^+^ CD4 T cells, in technical replicates for each plotted condition. Upon TCR stimulation, CD4 T cells significantly increased levels of CD200 and CD200R expression and co-express CD200∶CD200R (***p = 0.0005, NS = not significant), while IL-7-treated cells did not significantly differ from unstimulated controls. An independent biological replicate of Fig. 1A is presented. B. At an early time point (d3), CD4 T cells showed similar activation under all stimulation conditions. Naïve peripheral LN cells were transiently or chronically stimulated (as in Fig 1A) with anti-CD3 (1 µg/ml) + anti-CD28 (2 µg/ml), as indicated, in Th1 (top row), Th2 (middle row) or non-polarising (IL-7, bottom row) conditions. Histograms (unfilled) show expression of activation markers CD25 (left columns) and CD44 (right columns) compared to naïve controls (filled histograms) in CD4 gated T cells.
(TIF)

Figure S2 **CD4 T cells co-express CD200 and CD200R upon chronic activation.** A. Co-expression of CD200R and CD200 is shown from the experiment in Fig. 1C. Naïve LN cells were transiently (top rows) or chronically (bottom rows) stimulated with anti-CD3 (1 µg/ml) + anti-CD28 (2 µg/ml) in Th1, Th2, or non-polarising (IL-7) conditions. Contour plots show co-expression of CD200 and CD200R in CD4 gated T cells at d3 (top panels) and d7 (bottom panels) compared to the levels found in naïve CD4 T cells (left end column). Chronic TCR stimulation favoured co-expression of CD200 and CD200R and, by d7, the percentage of CD200^+^ CD200R^+^ CD4 T cells was significantly higher in chronic rather than transient stimulations, compared using a paired 2-tailed t-test (p<0.05). Additionally, CD200∶CD200R co-expressing CD4 cells significantly increased from d3 to d7 in chronically stimulated conditions (paired 2-tailed t-test, p<0.05). B. Co-expression of CD200∶CD200R occured primarily on activated CD25+CD44+ CD4 T cells. As an example, T cells cultured with chronic TCR stimulation for 7d under Th1 conditions are shown for expression of activation markers, CD44 and CD25. Th1 activated cells (left overlay, black dots) upregulated both CD44 and CD25 compared to naïve controls (left overlay, blue dots). 74% of CD44+ CD25+ CD4 T cells co-expressed CD200∶CD200R (red dots in right overlay). Equivalent profiles were obtained in any other chronic condition.
(TIF)

Figure S3 **Specificity of intracellular cytokine staining and significant upregulation of CD200R expression following transient versus chronic TCR stimulation.** A-B. Control staining (not stimulated with PdbU + iono) is shown for the intracellular cytokine stains presented in Fig. 2. In the absence of restimulation very little background cytokine staining was observed. C. Bar graph shows the percentage of CD200R^+^ CD4^+^ T cells in transient (n = 11, grey) and chronic (n = 11, black) TCR stimulations (Mean±SEM) compared with naïve (n = 4, white) controls, evaluated across all polarising conditions in 4 biological repeats of experiments shown in Fig. 1C and 2. CD200R up-regulation compared to naïve controls is shown following transient stimulation (4.83±0.83 to 21.9±3.4, *p = 0.01) and chronic stimulation (4.83±0.83 to 55.0±6.84, **p = 0.001) and between transient and chronic stimulation conditions (*p = 0.01, unpaired t-test). D. Shows 4 pooled biological repeats (n = 11) comparing the percentage of CD200R^+^ CD4 T cells under chronic and transient TCR stimulation linked by experiment under all polarising condition (*p = 0.01, n = 11, paired t-test). E. The percentage of CD200R^neg^ IL-2^+^ (n = 11) and F. CD200R^neg^ TNFα^+^ (n = 8) in transient compared to chronic TCR stimulations linked by experiment from 4 pooled biological repeats is shown. Upon chronic stimulation, CD200R^neg^ IL-2^+^ (E) and TNFα^+^ (F) CD4 T cells decreased significantly (***p = 0.0001 and **p = 0.001).
(TIF)

Figure S4 **Chronic infection causes an increase in activated CD4 T cells that co-express CD200**∶**CD200R.** C57Bl/6 mice were infected with *S. mansoni* and MesLN were analyzed. A. Graph shows the percentage (mean±SD) of activated/memory-phenotype, CD44^hi^ CD4 T cells in infected mice (n = 17) compared to naïve, uninfected controls (n = 14) 8 weeks after infection. CD44^hi^CD4 cells increased from 12.6±2.34% (n = 14) to 31.9±3.93% (n = 17, ***p = 4.86×10^–16^, 2-tailed, unpaired t-test). B. The percentage (mean±SD) of CD200^+^CD200R^+^CD4 cells in infected mice (n = 17) compared to naïve un-infected controls (n = 14) is shown. Upon infection, CD4 cells co-expressing CD200 and CD200R increased significantly from 7.82±3.52% (n = 14) to 19.3±7.72% (n = 17, ***p = 1.70×10^–5^, 2-tails, unpaired t-test). Data are pooled from the same 4 biological repeats shown in Fig. 3.
(TIF)

Figure S5 **Dying cells express very high levels of CD200R.** A. Contour plots show expression of CD44 and CD200R in total compared to viable CD4 T cells, based on forward and side scatter discrimination. Total CD4 T cells (left) show two populations of CD200R staining (R1 and R2), which disappear once dead cells are excluded in the viable gate (right). One example out of with 17 mice infected with *S. mansoni* from 4 independent experiments is shown. Specificity of intracellular IL-4 and GFP staining. B-C. An independent biological repeat of Fig. 3F shows the specificity of GFP detection in infected 4-get mice. B. No signal is dected in the GFP channel in intact cells from either *S. mansoni* infected wild-type C57BL/6 mice or naïve uninfected 4-get control mice. Upon infection, GFP^+^ CD4 T cells increased and homogenously upregulated CD200 expression in infected 4-get, but not C57BL/6 mice. C. Specificity of GFP signal is shown with indirect GFP detection using anti-GFP mAb in intracellular cytokine staining together with anti-IL-4, as in Fig. 2. In unstimulated controls (bottom panels, -P/I), GFP^+^ CD4 cells were only detected in infected 4-get mice and did not actively secrete IL-4. Upon 5h-restimulation in the presence of PdbU and Ionomycin (top panels, +P/I), IL-4^+^ CD4 cells detected in infected mice were GFP^+^ in 4-get, but not control C57BL/6 mice.
(TIF)

Figure S6 **CD4 T cells in** ***S. mansoni*** **infected animals proliferate and accumulate while upregulating CD200R.** C57BL/6 mice were infected with *S. mansoni* and received BrdU (i.p. and orally) 4 days before analysis. BrdU incorporation was measured in the MesLN (A–D) and the spleen (D) as described in Materials and Methods. A-B. Contour plots show specificity of BrdU staining (A. anti-BrdU mAb, clone 3D4, B. isotype control) with expression of CD44 (left panels) or CD200R (right panels) in uninfected (top row) and infected (bottom row) mice. C. Graphs show the percentage of activated/memory-phenotype (mean±SEM) CD44^hi^ (left) and CD200R^+^ (right) CD4 T cells incorporating BrdU compared to isotype control in infected (n = 4) and uninfected, naïve, mice (n = 4). Upon infection (8 wk), CD44^hi^BrdU^+^ and CD200R^+^BrdU^+^ CD4 cells increased significantly from 3.56±0.14% (n = 4) to 8.31±1.06% (n = 4, **p = 0.01, 2-tails, unpaired t-test) and from 1.55±0.0.06% (n = 4) to 5.44±0.6% (n = 4, ***p = 0.001, 2-tails, unpaired t-test), respectively. D. Graphs show absolute numbers (±SD) of CD200R^+^BrdU^+^ CD4 cells in mesenteric LN (left) and spleen (right), after background subtraction based on isotype controls (B-C). Upon infection, CD200R^+^BrdU^+^ CD4 cells accumulated significantly in the mesLN between naïve and infected mice, from (5.61±0.74)×10^4^ (n = 4) to (35.4±14.5)×10^4^ (n = 4, *p = 0.05) and, in the spleen, from (35.9±10.9)×10^4^ (n = 4) to (300±192)×10^4^ (n = 4, *p = 0.05). Independent biological repeat of Fig. 3E.
(TIF)

Figure S7 **Specificity of CD200 and CD200R mAb staining.** A. Dot plots show staining of FITC-labeled Rat IgG2a isotype control (left) or anti-CD200R mAb (right) together with expression of activation marker, CD44, in mouse CD4 T cells. B. Dot plots show staining of APC-labeled Rat IgG2a isotype control (left) or anti-CD200 mAb (right) together with expression of activation marker, CD44, in mouse CD4 T cells. In the upper left quadrants, the average±SD frequency of CD44hi CD200R^+^ (A) or CD200^+^ (B) CD4 T cells is indicated (n = 3 biological replicates), showing increased detection of CD200R and CD200 in memory-phenotype cells over the relative isotype controls.
(TIF)

Figure S8 **CD200R expression in vivo correlates inversely with TNFα and IL-2 cytokine production.** Bar graphs show (mean±SEM) detection of A. TNFα and B. IL-2 cytokine^+^ CD200R^neg^ CD4 cells *ex vivo* after recall with PdbU + Ionomycin (+P/I, 5h) as shown in Fig.4. A. Infection with *S. mansoni* (8wk, left) or *S. enterica* (2wk, right) decreased CD200R^neg^TNFα^+^ CD4 cells in mesLN from 35.6±4.4% to 18.1±3.1% (*p = 0.01, n = 10/group, data pooled from 3 independent biological repeats) in *S. mansoni* infection and in spleen in *S. enterica* infection from 32.6±5.2% to 12.3±1.4% (**p = 0.001, n = 10/group, data pooled from 2 independent biological repeats). B. In agreement with the loss of TNFα, infection also decreased CD200R^neg^IL-2^+^ CD4 cells in LN (*S. mansoni* at 8wks) from 34.2±1.67% to 21.0±1.37% (**p = 0.001, n = 4/group) and in spleen (*S. enterica* at 2wks) from 23.5±0.95% to 0.82±0.0.16% (***p = 0.0001, n = 5/group).
(TIF)

Figure S9 **Expression of CD200R in human CD45RA^–^ CD4^+^ αβTCR^+^ cells and isotype controls for intracellular stains.** A. Dot plots show expression of CD45RA and CD200R mAb (left), compared to isotype control stains (mouse IgG1, right), in gated αβTCR^+^ CD4^+^ lymphocytes from human PBMC. As previously found in Rijkers et al. (14), memory-phenotype (CD45RA^–^) CD4 T cells specifically up-regulated CD200R expression. B. Dot plot shows the isotype control stains for IL-4/IFNγ (y-axis) and CD200R (x-axis) mAbs used for the intracellular staining of human CD4^+^ T cells in Fig. 5A-B. C. Dot plots show intracellular stain of GATA-3 (right panel) versus an isotype control stain (left panel) after gating on CD4^+^ T cells, as used in Fig. 5C.
(TIF)
