# Supplementary figures and images for: Chronic Infection Drives Expression of the Inhibitory Receptor CD200R, and Its Ligand CD200, by Mouse and Human CD4 T Cells
Source: PLoS One. 2012 Apr 9;7(4):e35466. doi: 10.1371/journal.pone.0035466 (PMC3322173; doi:10.1371/journal.pone.0035466)

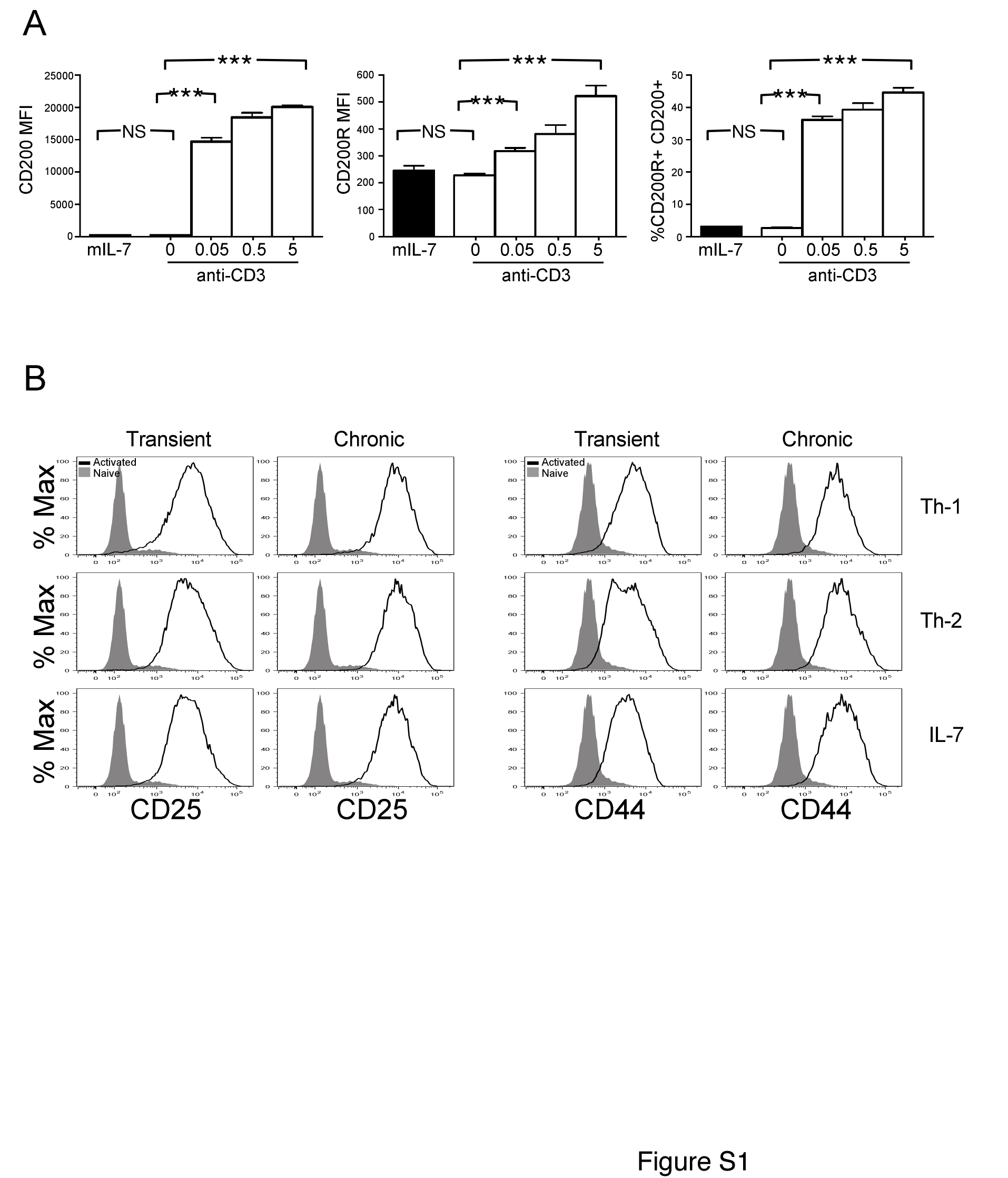

Supplement: Figure S1 — T cells co-express CD200 and CD200R following TCR stimulation. A. Naïve C57BL/6 peripheral LN cells were cultured with a titration of anti-CD3 + anti-CD28 (2 µg/ml) (white bars) or only with recombinant mouse IL-7 (10 ng/ml, black bar) for 3d. Bar graphs show average±SEM Mean Fluorescence Intensity (MFI) expression of CD200 (left) and CD200R (middle) in CD4 T cells and percentage of CD200+ CD200R+ CD4 T cells, in technical replicates for each plotted condition. Upon TCR stimulation, CD4 T cells significantly increased levels of CD200 and CD200R expression and co-express CD200∶CD200R (***p = 0.0005, NS = not significant), while IL-7-treated cells did not significantly differ from unstimulated controls. An independent biological replicate of Fig. 1A is presented. B. At an early time point (d3), CD4 T cells showed similar activation under all stimulation conditions. Naïve peripheral LN cells were transiently or chronically stimulated (as in Fig 1A) with anti-CD3 (1 µg/ml) + anti-CD28 (2 µg/ml), as indicated, in Th1 (top row), Th2 (middle row) or non-polarising (IL-7, bottom row) conditions. Histograms (unfilled) show expression of activation markers CD25 (left columns) and CD44 (right columns) compared to naïve controls (filled histograms) in CD4 gated T cells. (TIF) [file pone.0035466.s001.tif]

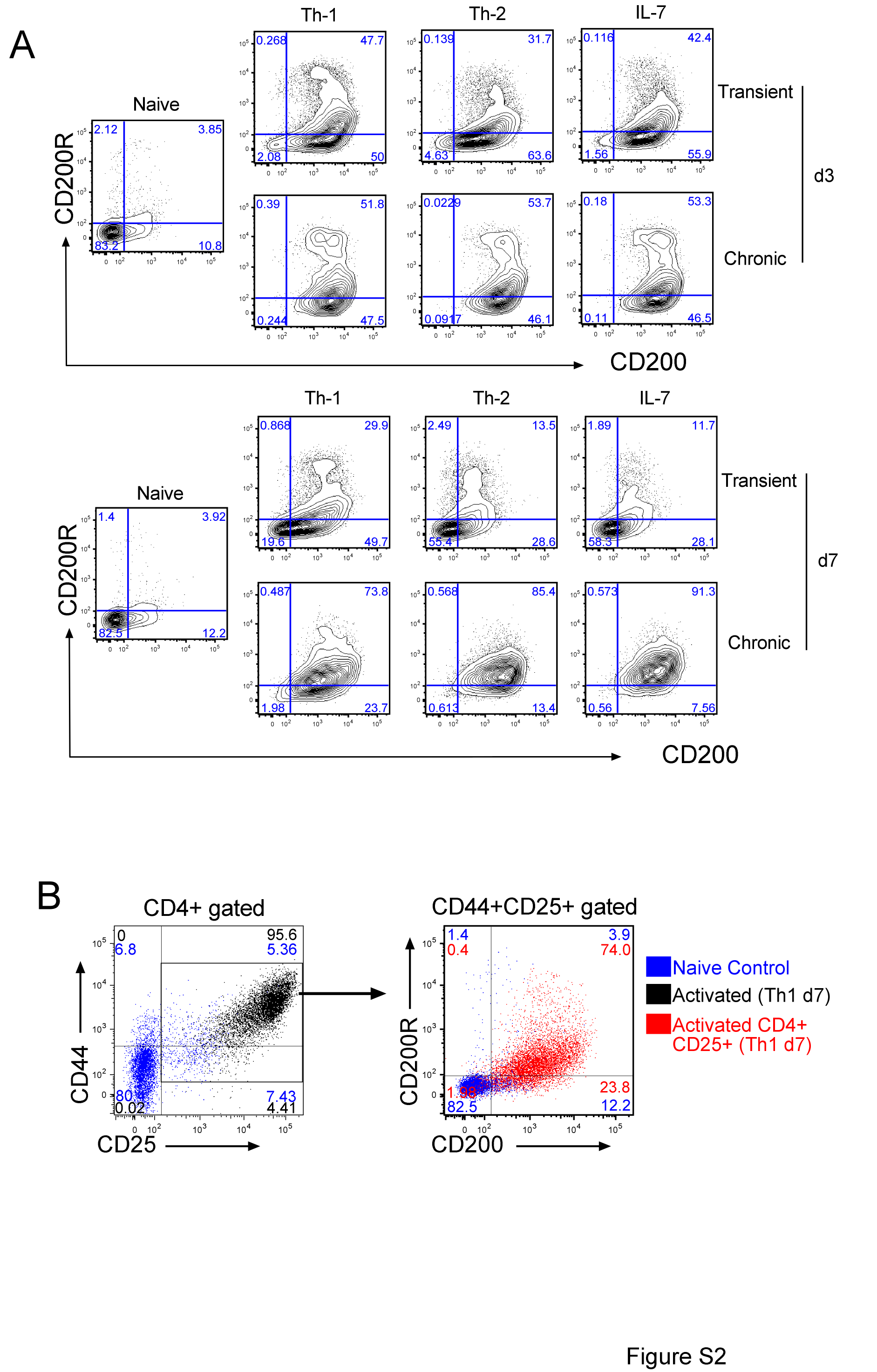

Supplement: Figure S2 — CD4 T cells co-express CD200 and CD200R upon chronic activation. A. Co-expression of CD200R and CD200 is shown from the experiment in Fig. 1C. Naïve LN cells were transiently (top rows) or chronically (bottom rows) stimulated with anti-CD3 (1 µg/ml) + anti-CD28 (2 µg/ml) in Th1, Th2, or non-polarising (IL-7) conditions. Contour plots show co-expression of CD200 and CD200R in CD4 gated T cells at d3 (top panels) and d7 (bottom panels) compared to the levels found in naïve CD4 T cells (left end column). Chronic TCR stimulation favoured co-expression of CD200 and CD200R and, by d7, the percentage of CD200+ CD200R+ CD4 T cells was significantly higher in chronic rather than transient stimulations, compared using a paired 2-tailed t-test (p<0.05). Additionally, CD200∶CD200R co-expressing CD4 cells significantly increased from d3 to d7 in chronically stimulated conditions (paired 2-tailed t-test, p<0.05). B. Co-expression of CD200∶CD200R occured primarily on activated CD25+CD44+ CD4 T cells. As an example, T cells cultured with chronic TCR stimulation for 7d under Th1 conditions are shown for expression of activation markers, CD44 and CD25. Th1 activated cells (left overlay, black dots) upregulated both CD44 and CD25 compared to naïve controls (left overlay, blue dots). 74% of CD44+ CD25+ CD4 T cells co-expressed CD200∶CD200R (red dots in right overlay). Equivalent profiles were obtained in any other chronic condition. (TIF) [file pone.0035466.s002.tif]

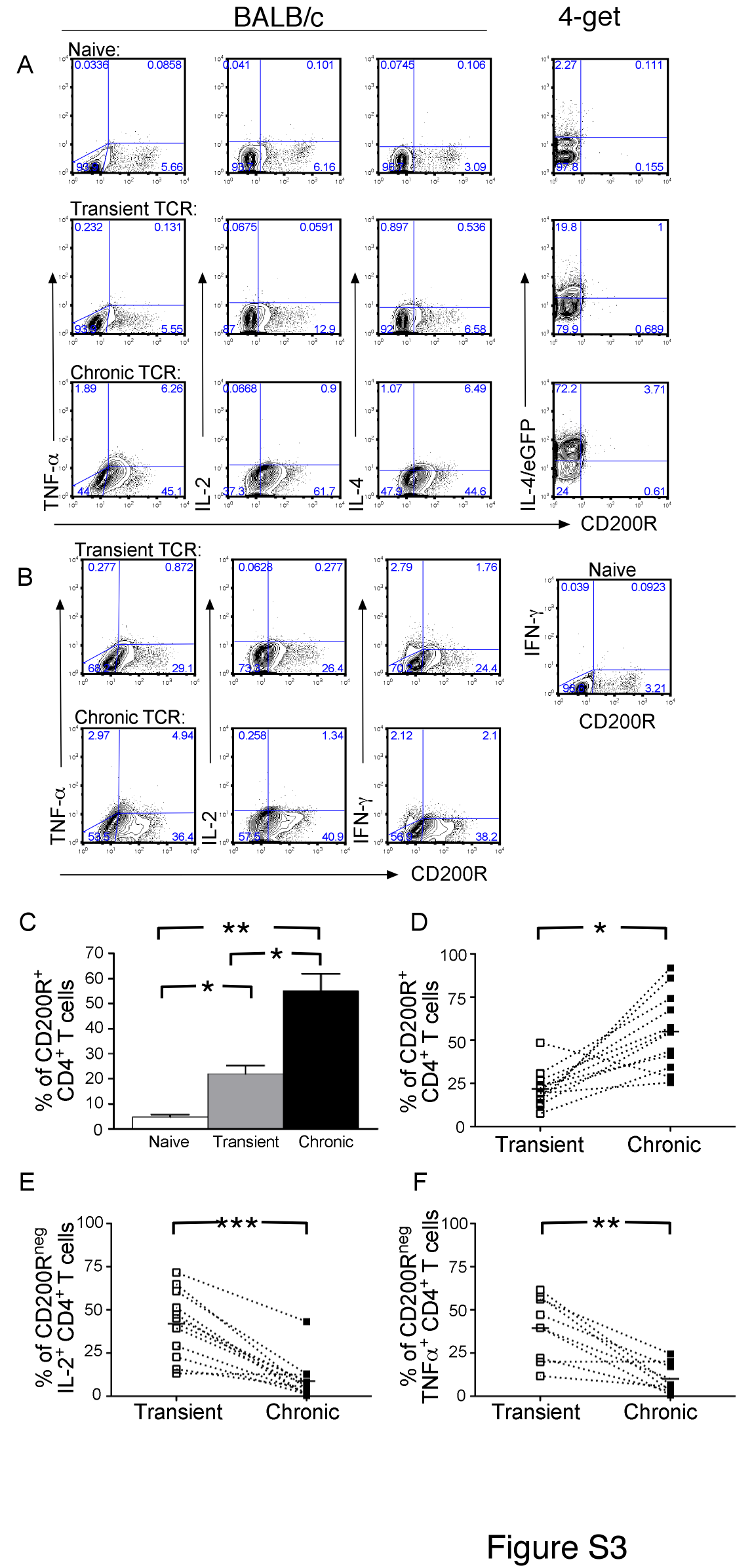

Supplement: Figure S3 — Specificity of intracellular cytokine staining and significant upregulation of CD200R expression following transient versus chronic TCR stimulation. A-B. Control staining (not stimulated with PdbU + iono) is shown for the intracellular cytokine stains presented in Fig. 2. In the absence of restimulation very little background cytokine staining was observed. C. Bar graph shows the percentage of CD200R+ CD4+ T cells in transient (n = 11, grey) and chronic (n = 11, black) TCR stimulations (Mean±SEM) compared with naïve (n = 4, white) controls, evaluated across all polarising conditions in 4 biological repeats of experiments shown in Fig. 1C and 2. CD200R up-regulation compared to naïve controls is shown following transient stimulation (4.83±0.83 to 21.9±3.4, *p = 0.01) and chronic stimulation (4.83±0.83 to 55.0±6.84, **p = 0.001) and between transient and chronic stimulation conditions (*p = 0.01, unpaired t-test). D. Shows 4 pooled biological repeats (n = 11) comparing the percentage of CD200R+ CD4 T cells under chronic and transient TCR stimulation linked by experiment under all polarising condition (*p = 0.01, n = 11, paired t-test). E. The percentage of CD200Rneg IL-2+ (n = 11) and F. CD200Rneg TNFα+ (n = 8) in transient compared to chronic TCR stimulations linked by experiment from 4 pooled biological repeats is shown. Upon chronic stimulation, CD200Rneg IL-2+ (E) and TNFα+ (F) CD4 T cells decreased significantly (***p = 0.0001 and **p = 0.001). (TIF) [file pone.0035466.s003.tif]

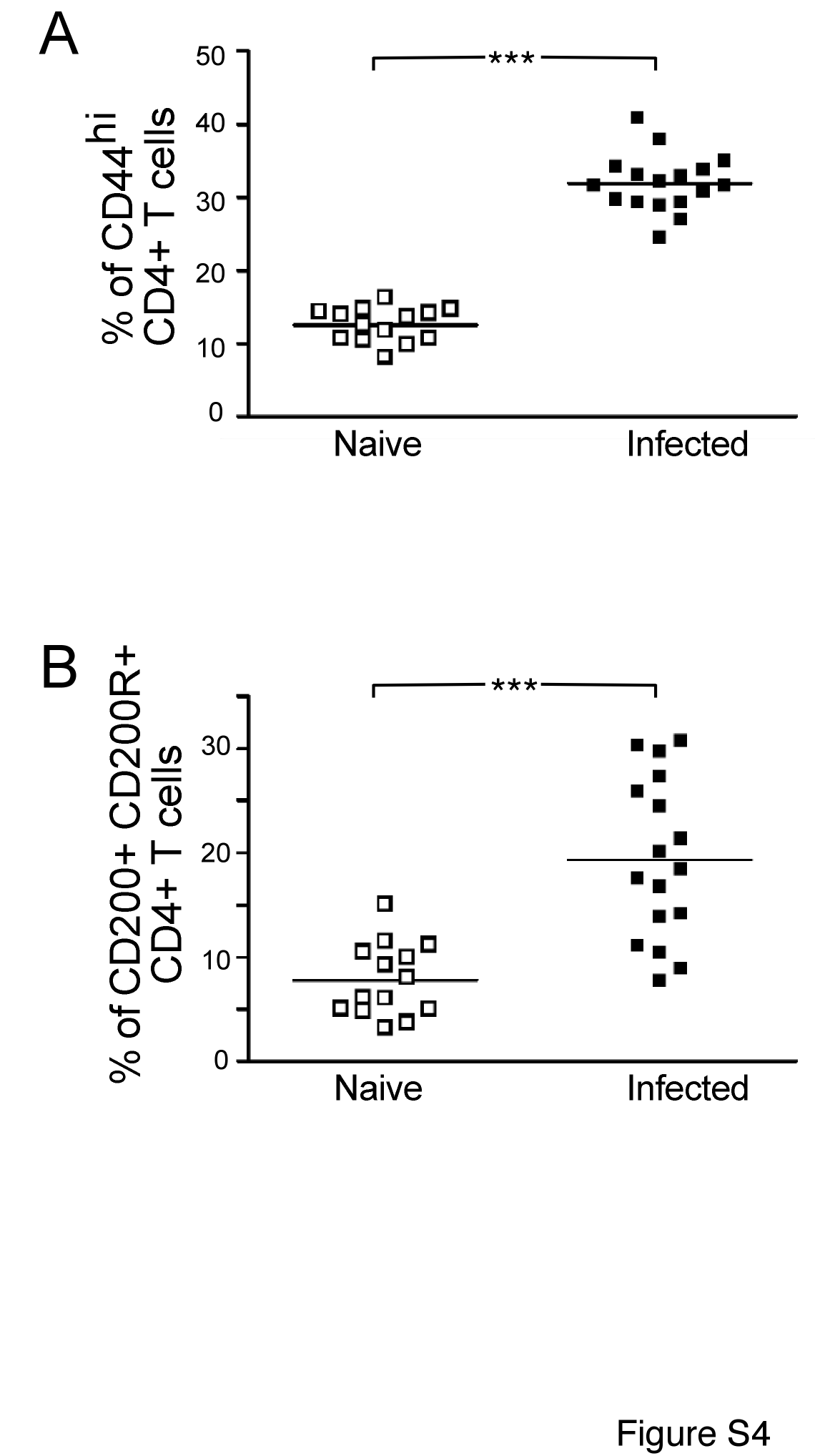

Supplement: Figure S4 — Chronic infection causes an increase in activated CD4 T cells that co-express CD200∶CD200R. C57Bl/6 mice were infected with S. mansoni and MesLN were analyzed. A. Graph shows the percentage (mean±SD) of activated/memory-phenotype, CD44hi CD4 T cells in infected mice (n = 17) compared to naïve, uninfected controls (n = 14) 8 weeks after infection. CD44hiCD4 cells increased from 12.6±2.34% (n = 14) to 31.9±3.93% (n = 17, ***p = 4.86×10^–16^, 2-tailed, unpaired t-test). B. The percentage (mean±SD) of CD200+CD200R+CD4 cells in infected mice (n = 17) compared to naïve un-infected controls (n = 14) is shown. Upon infection, CD4 cells co-expressing CD200 and CD200R increased significantly from 7.82±3.52% (n = 14) to 19.3±7.72% (n = 17, ***p = 1.70×10^–5^, 2-tails, unpaired t-test). Data are pooled from the same 4 biological repeats shown in Fig. 3. (TIF) [file pone.0035466.s004.tif]

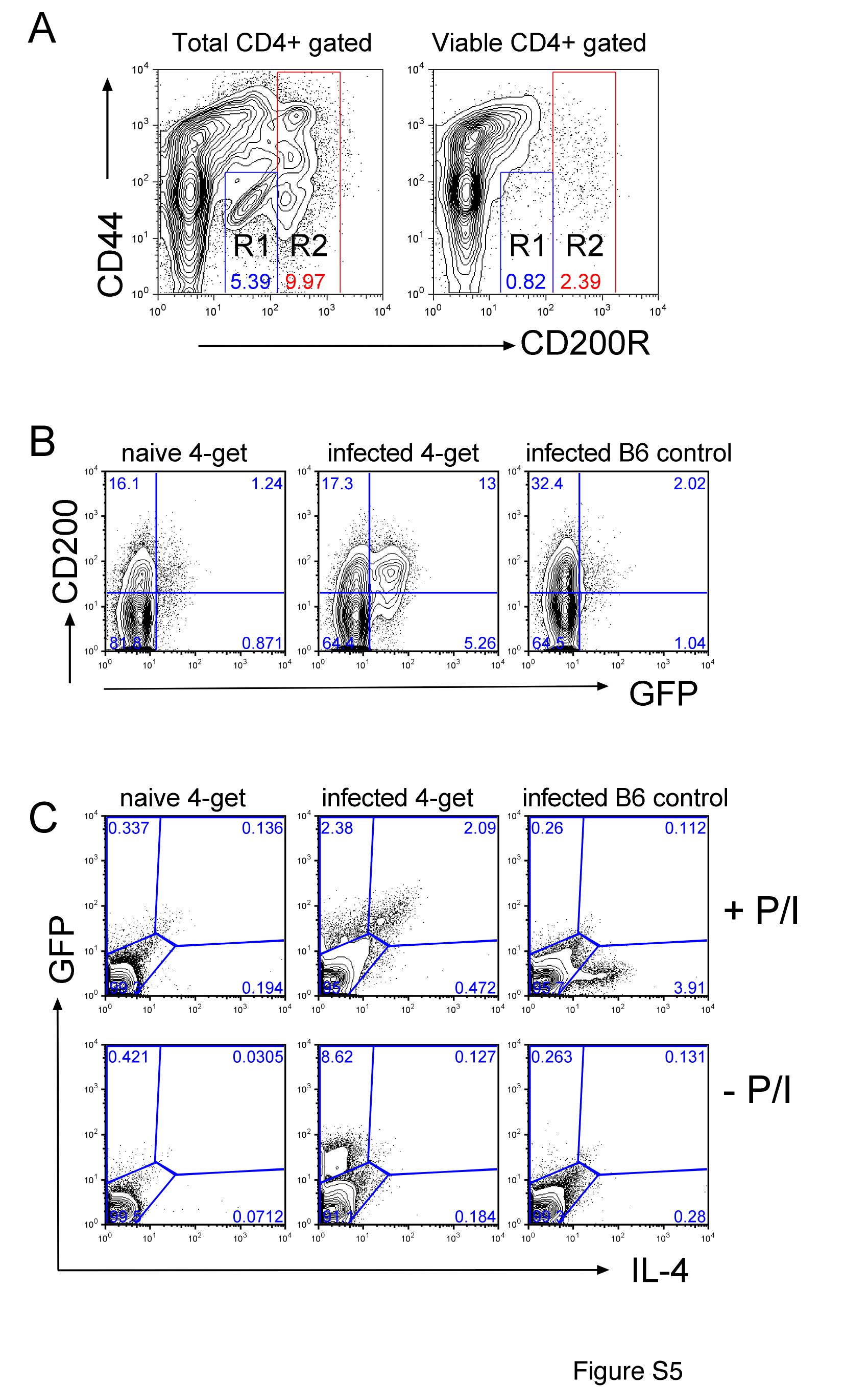

Supplement: Figure S5 — Dying cells express very high levels of CD200R. A. Contour plots show expression of CD44 and CD200R in total compared to viable CD4 T cells, based on forward and side scatter discrimination. Total CD4 T cells (left) show two populations of CD200R staining (R1 and R2), which disappear once dead cells are excluded in the viable gate (right). One example out of with 17 mice infected with S. mansoni from 4 independent experiments is shown. Specificity of intracellular IL-4 and GFP staining. B-C. An independent biological repeat of Fig. 3F shows the specificity of GFP detection in infected 4-get mice. B. No signal is dected in the GFP channel in intact cells from either S. mansoni infected wild-type C57BL/6 mice or naïve uninfected 4-get control mice. Upon infection, GFP+ CD4 T cells increased and homogenously upregulated CD200 expression in infected 4-get, but not C57BL/6 mice. C. Specificity of GFP signal is shown with indirect GFP detection using anti-GFP mAb in intracellular cytokine staining together with anti-IL-4, as in Fig. 2. In unstimulated controls (bottom panels, -P/I), GFP+ CD4 cells were only detected in infected 4-get mice and did not actively secrete IL-4. Upon 5h-restimulation in the presence of PdbU and Ionomycin (top panels, +P/I), IL-4+ CD4 cells detected in infected mice were GFP+ in 4-get, but not control C57BL/6 mice. (TIF) [file pone.0035466.s005.tif]

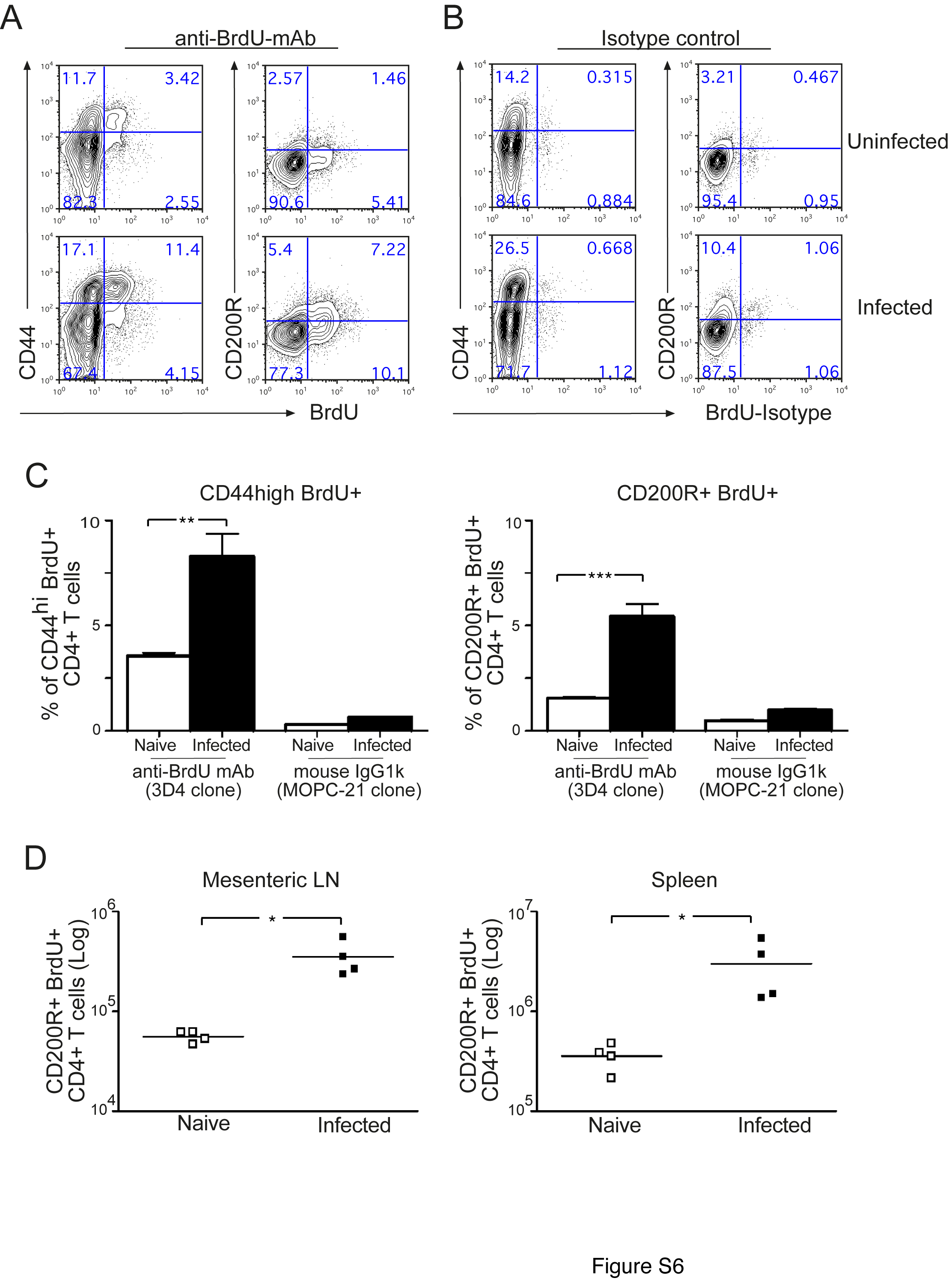

Supplement: Figure S6 — CD4 T cells in S. mansoni infected animals proliferate and accumulate while upregulating CD200R. C57BL/6 mice were infected with S. mansoni and received BrdU (i.p. and orally) 4 days before analysis. BrdU incorporation was measured in the MesLN (A–D) and the spleen (D) as described in Materials and Methods. A-B. Contour plots show specificity of BrdU staining (A. anti-BrdU mAb, clone 3D4, B. isotype control) with expression of CD44 (left panels) or CD200R (right panels) in uninfected (top row) and infected (bottom row) mice. C. Graphs show the percentage of activated/memory-phenotype (mean±SEM) CD44hi (left) and CD200R+ (right) CD4 T cells incorporating BrdU compared to isotype control in infected (n = 4) and uninfected, naïve, mice (n = 4). Upon infection (8 wk), CD44hiBrdU+ and CD200R+BrdU+ CD4 cells increased significantly from 3.56±0.14% (n = 4) to 8.31±1.06% (n = 4, **p = 0.01, 2-tails, unpaired t-test) and from 1.55±0.0.06% (n = 4) to 5.44±0.6% (n = 4, ***p = 0.001, 2-tails, unpaired t-test), respectively. D. Graphs show absolute numbers (±SD) of CD200R+BrdU+ CD4 cells in mesenteric LN (left) and spleen (right), after background subtraction based on isotype controls (B-C). Upon infection, CD200R+BrdU+ CD4 cells accumulated significantly in the mesLN between naïve and infected mice, from (5.61±0.74)×104 (n = 4) to (35.4±14.5)×104 (n = 4, *p = 0.05) and, in the spleen, from (35.9±10.9)×104 (n = 4) to (300±192)×104 (n = 4, *p = 0.05). Independent biological repeat of Fig. 3E. (TIF) [file pone.0035466.s006.tif]

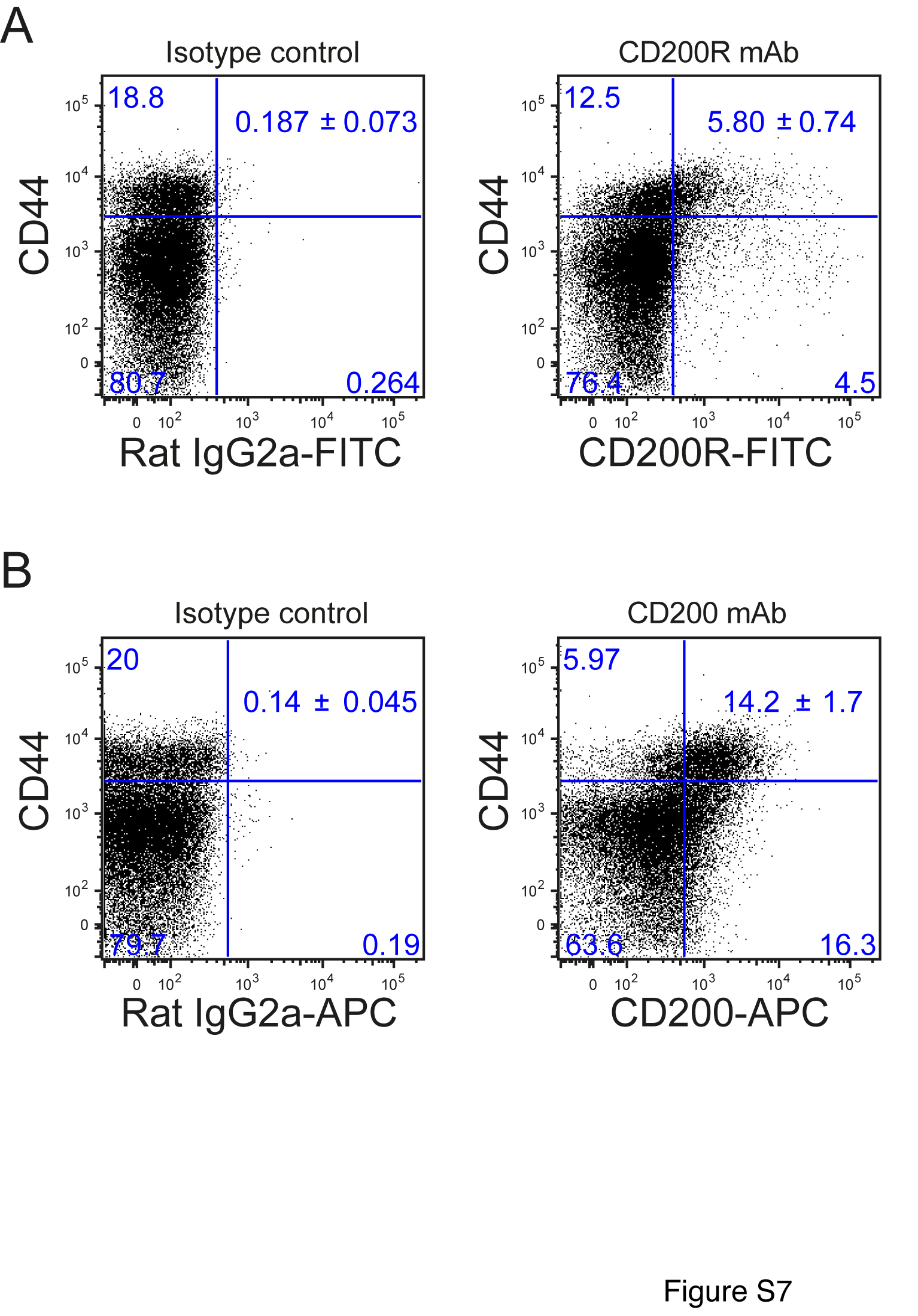

Supplement: Figure S7 — Specificity of CD200 and CD200R mAb staining. A. Dot plots show staining of FITC-labeled Rat IgG2a isotype control (left) or anti-CD200R mAb (right) together with expression of activation marker, CD44, in mouse CD4 T cells. B. Dot plots show staining of APC-labeled Rat IgG2a isotype control (left) or anti-CD200 mAb (right) together with expression of activation marker, CD44, in mouse CD4 T cells. In the upper left quadrants, the average±SD frequency of CD44hi CD200R+ (A) or CD200+ (B) CD4 T cells is indicated (n = 3 biological replicates), showing increased detection of CD200R and CD200 in memory-phenotype cells over the relative isotype controls. (TIF) [file pone.0035466.s007.tif]

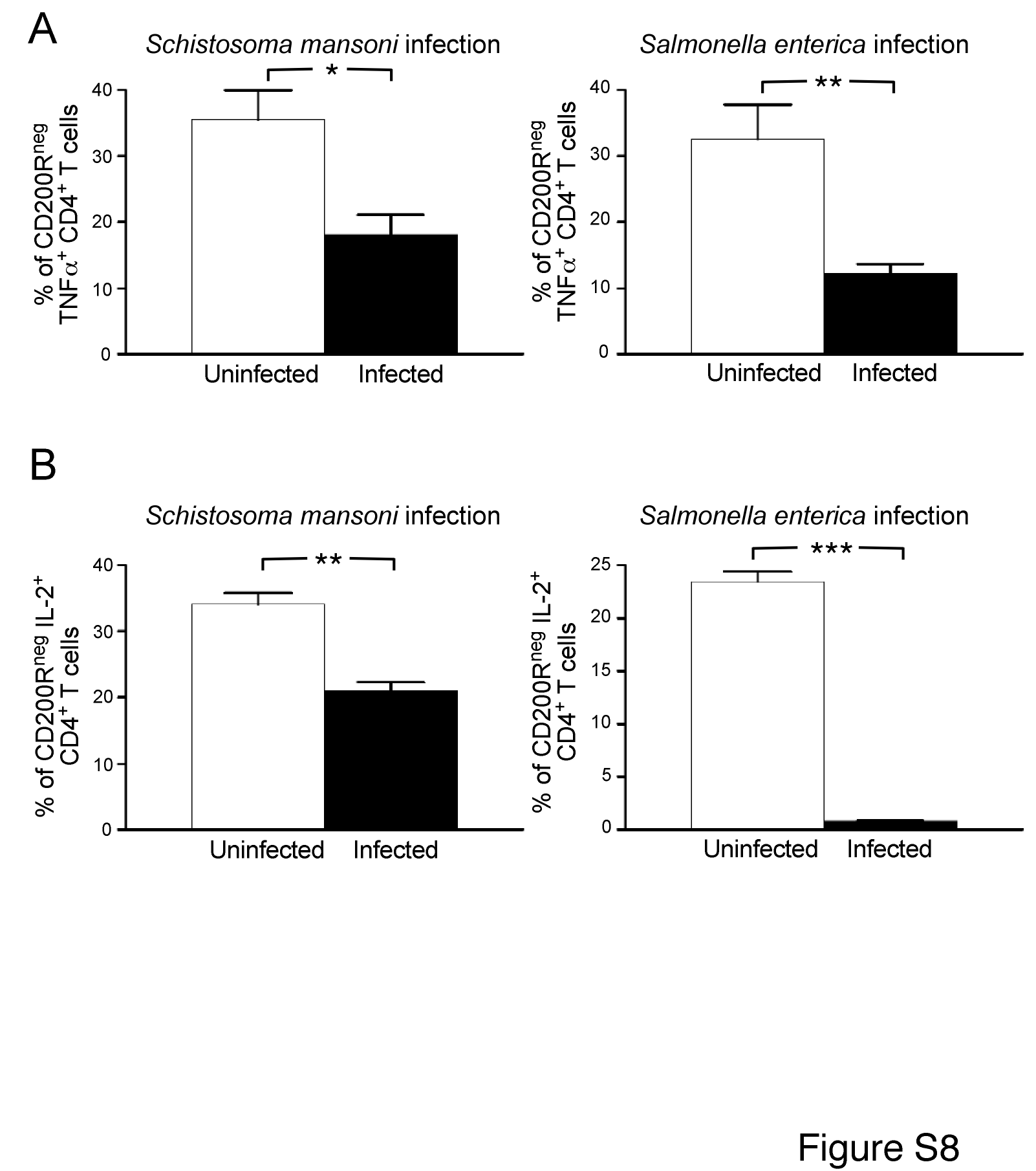

Supplement: Figure S8 — CD200R expression in vivo correlates inversely with TNFα and IL-2 cytokine production. Bar graphs show (mean±SEM) detection of A. TNFα and B. IL-2 cytokine+ CD200Rneg CD4 cells ex vivo after recall with PdbU + Ionomycin (+P/I, 5h) as shown in Fig.4. A. Infection with S. mansoni (8wk, left) or S. enterica (2wk, right) decreased CD200RnegTNFα+ CD4 cells in mesLN from 35.6±4.4% to 18.1±3.1% (*p = 0.01, n = 10/group, data pooled from 3 independent biological repeats) in S. mansoni infection and in spleen in S. enterica infection from 32.6±5.2% to 12.3±1.4% (**p = 0.001, n = 10/group, data pooled from 2 independent biological repeats). B. In agreement with the loss of TNFα, infection also decreased CD200RnegIL-2+ CD4 cells in LN (S. mansoni at 8wks) from 34.2±1.67% to 21.0±1.37% (**p = 0.001, n = 4/group) and in spleen (S. enterica at 2wks) from 23.5±0.95% to 0.82±0.0.16% (***p = 0.0001, n = 5/group). (TIF) [file pone.0035466.s008.tif]

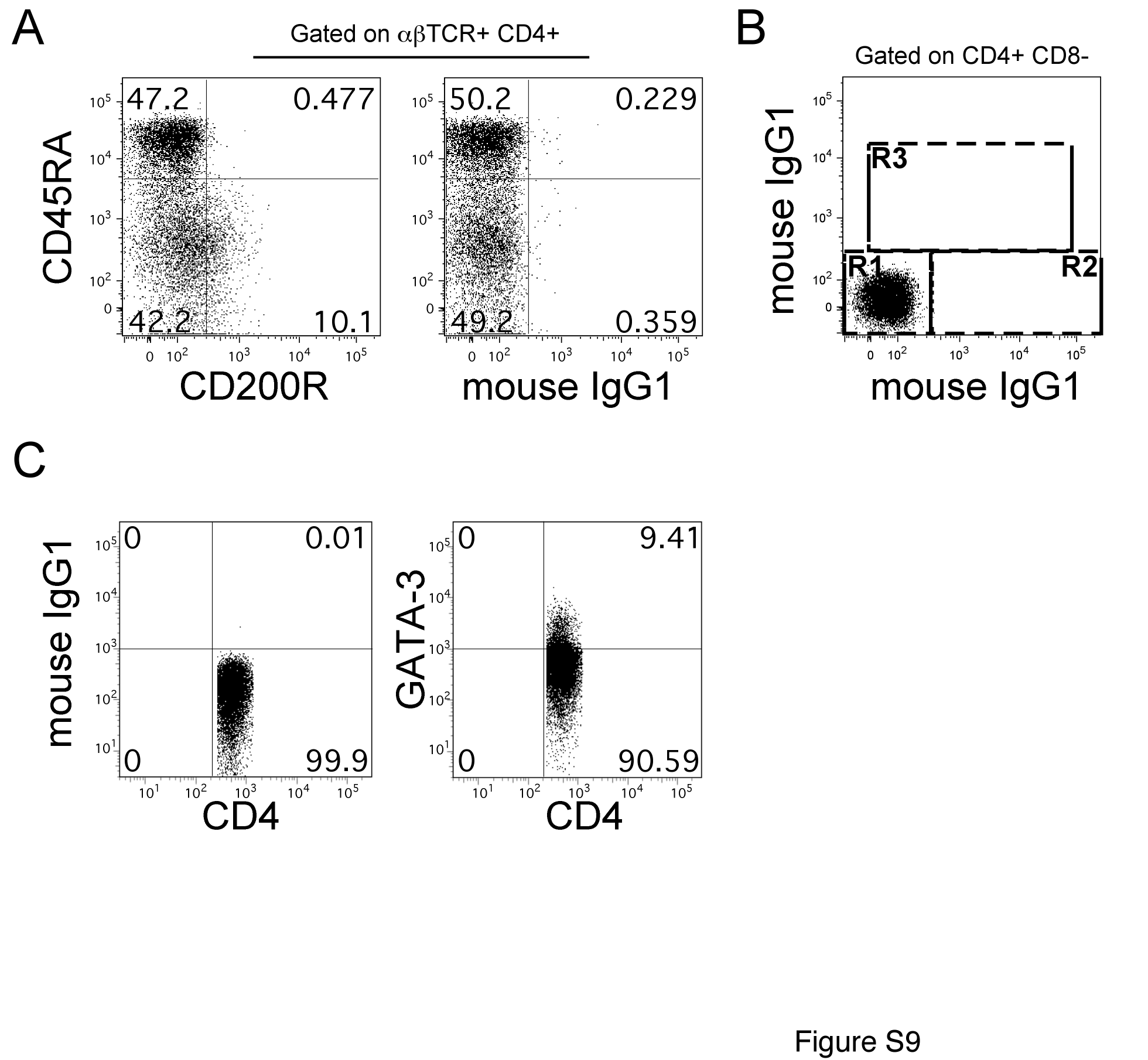

Supplement: Figure S9 — Expression of CD200R in human CD45RA– CD4+ αβTCR+ cells and isotype controls for intracellular stains. A. Dot plots show expression of CD45RA and CD200R mAb (left), compared to isotype control stains (mouse IgG1, right), in gated αβTCR+ CD4+ lymphocytes from human PBMC. As previously found in Rijkers et al. (14), memory-phenotype (CD45RA–) CD4 T cells specifically up-regulated CD200R expression. B. Dot plot shows the isotype control stains for IL-4/IFNγ (y-axis) and CD200R (x-axis) mAbs used for the intracellular staining of human CD4+ T cells in Fig. 5A-B. C. Dot plots show intracellular stain of GATA-3 (right panel) versus an isotype control stain (left panel) after gating on CD4+ T cells, as used in Fig. 5C. (TIF) [file pone.0035466.s009.tif]
